# Dietary Patterns and Fractures Risk in the Elderly

**DOI:** 10.3389/fendo.2017.00344

**Published:** 2017-12-13

**Authors:** Carmela Colica, Elisa Mazza, Yvelise Ferro, Antonietta Fava, Daniele De Bonis, Marta Greco, Daniela Patrizia Foti, Elio Gulletta, Stefano Romeo, Arturo Pujia, Tiziana Montalcini

**Affiliations:** ^1^Institute of Molecular Bioimaging and Physiology, CNR, Organizational Support Unit, University Magna Grecia, Catanzaro, Italy; ^2^Department of Medical and Surgical Science, Nutrition Unit, University Magna Grecia, Catanzaro, Italy; ^3^Department of Health Science, Laboratory Unit, University Magna Grecia, Catanzaro, Italy; ^4^Department of Molecular and Clinical Medicine, Sahlgrenska Center for Cardiovascular and Metabolic Research, University of Gothenburg, Göteborg, Sweden; ^5^Department of Clinical and Experimental Medicine, Nutrition Unit, University Magna Grecia, Catanzaro, Italy

**Keywords:** bone mineral density, principal components analysis, elderly, dietary patterns, meat, grains, olive oil

## Abstract

**Purpose:**

Although the role of dietary factors in the prevention of bone loss and fractures has been investigated in many studies, few studies have examined the association between dietary patterns and total body bone density. Our aim was to determine the relations between dietary patterns and whole-body bone mineral density (WB-BMD) and the association between dietary patterns, fractures, and multiple fractures in the elderly.

**Methods:**

This cross-sectional study included 177 individuals aged ≥65 years. A dual X-ray absorptiometry scan was performed to measure BMD. Dietary patterns were ascertained by a combination of dietary intake assessment and principal components analysis.

**Results:**

Only three dietary patterns correlated with whole-body bone density. The multivariate-adjusted mean bone density across tertiles of these dietary patterns showed that the highest tertile of both the patterns 1 and 2 had a significantly higher bone density than the lowest tertile (pattern 1: 1.021 ± 0.01 and 1.070 ± 0.01 g/cm^2^ for T1 and T3, respectively; *p* = 0.043; pattern 2: 1.023 ± 0.01, and 1.081 ± 0.01 g/cm^2^ for T1 and T3, respectively; *p* = 0.003). We also find significant gender difference in these results. The highest adherence to the dietary pattern 5 was associated with decreased odds of having fractures (OR = 0.20, *p* = 0.009), and adherence to the pattern 1 was negatively associated with multiple fractures.

**Conclusion:**

A high adherence to the dietary pattern 1 (high intake of grains, fish and olive oil) was associated with a high BMD and a low number of fractures. The highest adherence to the dietary pattern 5 (legumes and wine) was associated with decreased odds of having fractures. Our finding would suggest a potential bone-preserving properties of specific dietary patterns in the elderly.

## Introduction

The potential role of multiple dietary factors on bone health and fracture risk has been widely reported (1). Adherence to a healthy dietary pattern consisting in the intake of a high amount of fruit, vegetables, whole grains, fish, nuts, and legumes and low-fat dairy products decreases osteoporosis and fracture risk (1). It has been found that an increased intake of nutrient-dense foods (that provide a high amount of nutrients but have relatively few calories) together with a low intake of sweet foods is associated with a low risk for osteoporosis and fractures in adults and elderly individuals (2, 3). Diet is more related to fracture in older individuals (2), possibly since food choices matter more in older individuals (2). However, the relationship of nutrients–bone mass is complex and needs further in-depth investigation. For instance, it is not clear whether whole grains, fruits, and vegetables have beneficial effects on bone due to the alkaline ash these foods create or direct effect of the minerals on bone cells. Studies testing the cumulative effects of a combination of nutrients on bone mineral density (BMD) seem to provide more realistic results than studies on the potential effect of an individual nutrient because such nutrient effects might be too small to be identify (4). In addition, the majority of studies have investigated the relationship between nutrients or dietary patterns and spine and/or hip BMD (1–3). It is well accepted, however, that there may be a discordance in the diagnosis of osteoporosis using spine and hip bone densitometry (5). Osteoarthritis in these two sites may have dramatic effects on BMD measurements in the elderly (6, 7). In particular, the effect of osteophytes may be sufficient enough to cause 20–50% of men and 10–25% of women to be misdiagnosed (7). Proximal femur fracture, implants or a difficult femur rotation due to arthritis or pain render BMD measurement inaccurate and may vary between skeletal sites, especially in the elderly (6). There is a close relationship between whole-body (WB)-BMD and that assessed by spine and hip scans (8) and between WB-BMD and fractures (9). However, few studies have investigated the association between dietary patterns and WB-BMD. While postmenopausal osteoporosis causes accelerated trabecular bone resorption and tends to involve the spine, femur, and wrist, senile osteoporosis affects both trabecular and cortical bone, resulting in characteristic fractures of the proximal humerus, tibia, pelvis, and hip (10), thus a WB-BMD assessment may be a precious methodology in the elderly. For these reasons, in this study the main measurement outcome was WB-BMD. The objective of the present study was to determine the relations between dietary patterns and WB-BMD and the possible association between dietary patterns and fractures and the number of fractures (multiple fractures) in a population of elderly individuals.

## Materials and Methods

This is a cross-sectional study conducted from February 2013 to June 2014, whose protocol was approved by the local ethics committee at the “Mater Domini” University Hospital in Catanzaro, Italy and approved by the Italian Ministry of Health (projects codes 2011.48). The participants were invited to participate in the study by newspapers advertisements. We enrolled white, community-dwelling individuals aged ≥65 years from Calabria, southern Italy. Subjects were excluded if they presented any clinical condition affecting bone metabolism (such as kidney, liver, thyroid or parathyroids, rheumatic diseases, malabsorption syndromes, malignant tumors, or hematological diseases). No patients were taking dietary supplements, psychotropic drugs, glucocorticoids, estrogens, thyroid hormone, fluoride, or calcitonin as ascertained from their medical history, a physical examination, and laboratory tests (11, 12). Undergoing therapy for osteoporosis, hypertension, hyperlipidemia, and diabetes was not considered an exclusion criterion.

All participants underwent a WB-dual X-ray absorptiometry scan to measure WB-BMD. Written informed consent was obtained from participants. The investigation conforms to the principles outlined in the Declaration of Helsinki (13).

### Fractures Assessment

The prevalence of morphological vertebral fractures was determined by lateral thoracolumbar radiographs which were read by a radiologist. A vertebral fracture was quantitatively defined as a loss of height in the anterior, middle, or posterior dimension of the vertebral body that exceeded 20% relative to normal looking adjacent vertebra or relative to what one would expect normal vertebral height to be at that level (14). In addition to changes in dimension, vertebral fractures were detected on the basis of the presence of endplate deformities, the lack of parallelism of the endplates, and the general altered appearance compared with neighboring vertebrae (14).

Non-vertebral fractures were assessed through interviews or the evaluation of clinical records (when available). Fractures number was estimated for each participants. Pathological or high-energy fractures such as those caused by major trauma (e.g., motor vehicle accidents, a fall from a height higher than standing), and fractures in sites not commonly associated with osteoporosis such as those of the fingers, face, skull, and toes, were not taken into account.

### Dietary Intake Assessment

Dietary intake data were assessed by a 24-h recall and a 7-day food record, and calculated using nutritional software MetaDieta 3.0.1 (Metedasrl, San Benedetto del Tronto, Italy) (11). Participants ate freely and self-reported their intake. Precisely, the 24-h recall was performed *via* a face-to-face interview with a dietitian who used images associated with a comprehensive food list. The recall required 15–20 min to complete for each participant. The patients were also asked to report any ingredients, food and food waste in a food diary for a 7-day period. Each patient was trained by a skilled dietitian before starting the study. The dietitian showed how various foods should be recorded. The portion sizes used were based on the typical or natural portion consumed (e.g., a slice of bread, one egg). When a typical portion size was not obvious, a commonly used portion size was selected (e.g., one cup). We focused on major food groups, such as consumption of fish, meat, cereals, fruits, olive oil, wine, legumes, eggs, milk, potatoes, vegetables, sugary drinks, animal fats/margarines, and cake/pies. The database used to calculate energy intake was derived primarily from INRAN (National Institute of Food Research) 2000 and IEO (European Institute of Oncology) 2008 (11). This database includes over 5,000 foods and brand name products, and is updated annually.

### Assessment of the Factors and Medications Influencing BMD

We assessed the presence of the factors, such as diabetes, obesity, smoking, sufficient levels of physical activity (SPA), and medications use which may affect bone, from clinical records and patients interview (15, 16). Obesity was defined by the presence of a body mass index (BMI) ≥30 kg/m^2^ (17). Definition of SPA was the following: 150 min total of walking, moderate or vigorous PA per week (16).

### Biochemical Evaluation

Venous blood was collected after fasting overnight into vacutainer tubes (Becton & Dickinson, Plymouth, England) and centrifuged within 4 h. Serum glucose, creatinine, and calcium were measured with enzymatic colorimetric test. 25-hydroxy vitamin D—25OH D—was measured by radioimmunoassay (normal value 30–100 ng/mL). Quality control was assessed daily for all determinations.

### WB-BMD Measurement

Whole-body bone mineral density was measured using a dual X-ray absorptiometry (DXA) scan (Hologic QDR Inc., MA, USA). BMD was expressed as the amount of mineral (grams) divided by the area scanned (square centimeters) (12). Bone density was then expressed as the *T* score defined as the number of SDs from the healthy young adult mean while the *Z* score was the number of SDs in comparison to healthy subjects of the same age (12, 18). The DXA instrument was calibrated daily in accordance with the manufacturer’s recommendations. The *in vivo* precision was <1%.

## Statistical Analysis

Data are reported as mean ± SD. To find a 4% difference (~0.040 g/cm^2^ of BMD) between high and low tertiles of dietary pattern scores, considering a SD (*S*) of the outcome in the population equal to 0.07 and an effect size (*E*) equal to 0.04 with a *E*/*S* ratio equal to 0.60, with 80% power on a two-sided level of significance of 0.05, 44 subjects each group are required.

### Food Pattern Analysis

We obtained food patterns by using the principal components analysis (PCA) (11, 19). The goal of this analysis is to transform a given data set of large dimension to an alternative data set of smaller dimension. The food variables were correlated together (correlation coefficients *r* > 0.4). The orthogonal rotation (obtained with varimax option) was used to derive food patterns (non-correlated components). To fix the number of components to retain, a screen plot of the eigenvalues, that derived from the correlation matrix of the standardized variables, was examined. The eigenvalue is a value that indicates the proportion of the variance in consumption explained by each component. According to the Kaiser criterion, the number of components that should be retained from PCA is equal to the number of eigenvalues that are greater than 1. The food patterns were named according to scores of the foods that correlated most with the factor (>0.4). A higher absolute values in the correlation coefficients indicated that the food variable contributes most to the construction of the component. Principal components are the directions where there is the most variance. The eigenvector with the highest eigenvalue is therefore the principal components. Thus, the data set was reduced from large dimensions (the average consumption of single types of food in grams per person/day) to only six patterns by ignoring all the eigenvectors that have insignificant eigenvalues.

### Association between Dietary Patterns and WB-BMD

Pearson’s correlations were used to identify the dietary patterns (i.e., factors scores as continuous variables) and the variables (i.e., age, BMI, serum glucose, calcium, creatinine, and vitamin D) correlated with WB-BMD, given that the continuous variables were normally distributed. We also tested the correlations with the medications and fractures as binary variables.

Dietary pattern scores were categorized into tertiles (high, medium, and low levels) to provide sensitive indicators of the food intake level to assess the association of a food pattern intake to the WB-BMD. In the whole population, we compared the adjusted mean for each tertiles of each dietary pattern using the general linear model and performing two models. In model 1, we adjusted for BMI, glucose, and creatinine (according with the Pearson’s correlations); in model 2, we further adjusted for gender, medications (as antiosteoporotics, antihypertensives, anticoagulants, antidiabetics, statins, calcium, and vitamin D supplements), and current smoking. We also performed this statistical analysis by gender. *P* values to test for linear trends were calculated by using dietary pattern scores as a continuous variable after control for possible confounding factors.

### Association between Dietary Patterns, Fractures, and Number of Fractures

We classified the population in two groups according to the presence of fracture (i.e., individuals with vs. without fracture). Then, we used a multinomial logistic regression analysis to estimate the adjusted odds ratios (ORs) for the lowest and middle tertile of the dietary patterns intakes of having a fracture as compared with the highest tertile group (reference category).

Furthermore, we categorized the population according to the presence of “multiple fractures” (i.e., participants with only one fracture vs. those with more than one fracture). A binary logistic regression analysis was performed to test the association between all of the potentially confounding variables and dietary patterns (as independent variables), with “multiple fracture” serving as the dependent variable (potential confounders were all those factors correlated in the univariate analysis with a *p* value <0.1).

Significant differences were assumed to be present at *p* < 0.05 (two-tailed). All comparisons were performed using SPSS 20.0 for Windows (IBM Corporation, New York, NY, USA).

## Results

A total of 177 individuals (63% female) were enrolled. Table 1 shows the characteristics of the study population and Table 2 shows the food groups used in the PCA. The mean age was 69 ± 3 years. The mean energy intake was 1,867 ± 449 kcal (1,884 ± 478 in male and 1,858 ± 433 in female). A total of 41 participants (23%) had fractures (total fractures number = 52; femoral *n* = 1; vertebral *n* = 6; other sites *n* = 45). More than one fracture was reported in nine individuals. None of the participants had a SPA. In Table S1 in Supplementary Material, we report the characteristics of the study population by gender. A significant difference exists regarding age (Table S1 in Supplementary Material).

**Table 1:** Mean ± SD participants’ demographic, anthropometric, and clinical characteristics.

Variables	Mean	SD
Age (years)	70	4.1
MMSE	24	2
BMI (kg/m^2^)	29	4.1
SBP (mmHg)	133	16
DBP (mmHg)	80	9
Glucose (mg/dL)	102	24
Creatinine (mg/dL)	0.82	0.2
Calcium (mg/dL)	9	0.3
Vitamin D (nmol/L)	71	31
Vitamin D (ng/mL)	28	13
BMD (g/cm^2^)	1.04	0.1
Total body *T*-score	−1.08	1.3
Total body *Z*-score	−0.09	0.9

**Prevalence**		

Smokers (%)	12	
Lipid-lowering agents (%)	37	
Antihypertensive agents (%)	70	
Anticoagulant (%)	2	
Antiosteoporotic agents and calcium, vitamin D supplementation (%)	12	
Diabetes/carbohydrate intolerance (%)	23	
Oral antidiabetic agents (%)	12	
Sufficient level of physical (%)		
Fractures (%)	23	

*MMSE, mini mental state examination; BMI, body mass index; SBP, systolic blood pressure; DBP, diastolic blood pressure; BMD, bone mineral density*.

**Table 2:** Mean ± SD participants’ energy and food groups intake.

Food groups	Mean	SD
Cereals (g)^a^	109	46
Legumes (g)^a^	10	12
Potatoes (g)^a^	11	13
Vegetables (g)^a^	154	87
Fruit (g)^a^	189	113
Milk (g)^a^	70	62
Cheeses (g)^a^	34	26
Eggs (g)^a^	7	10
Meat (g)^a^	43	26
Fish (g)^a^	33	29
Wine (g)^a^	39	61
Sugary drinks (g)^a^	11	34
Olive oil (g)^a^	19	8
Animal-based fats/margarines (g)^a^	0.4	1.8
Cakes/pies (g)^a^	20	18
Energy intake (kcal) per day	1867	449
Carbohydrates (g/day)	216	59
Lipids(g/day)	72	22
Protein (g/day)	77	22

*^a^Adjusted for 1,000/kcal*.

### Food Patterns Analysis and Association with WB-BMD

**Figure 1:**
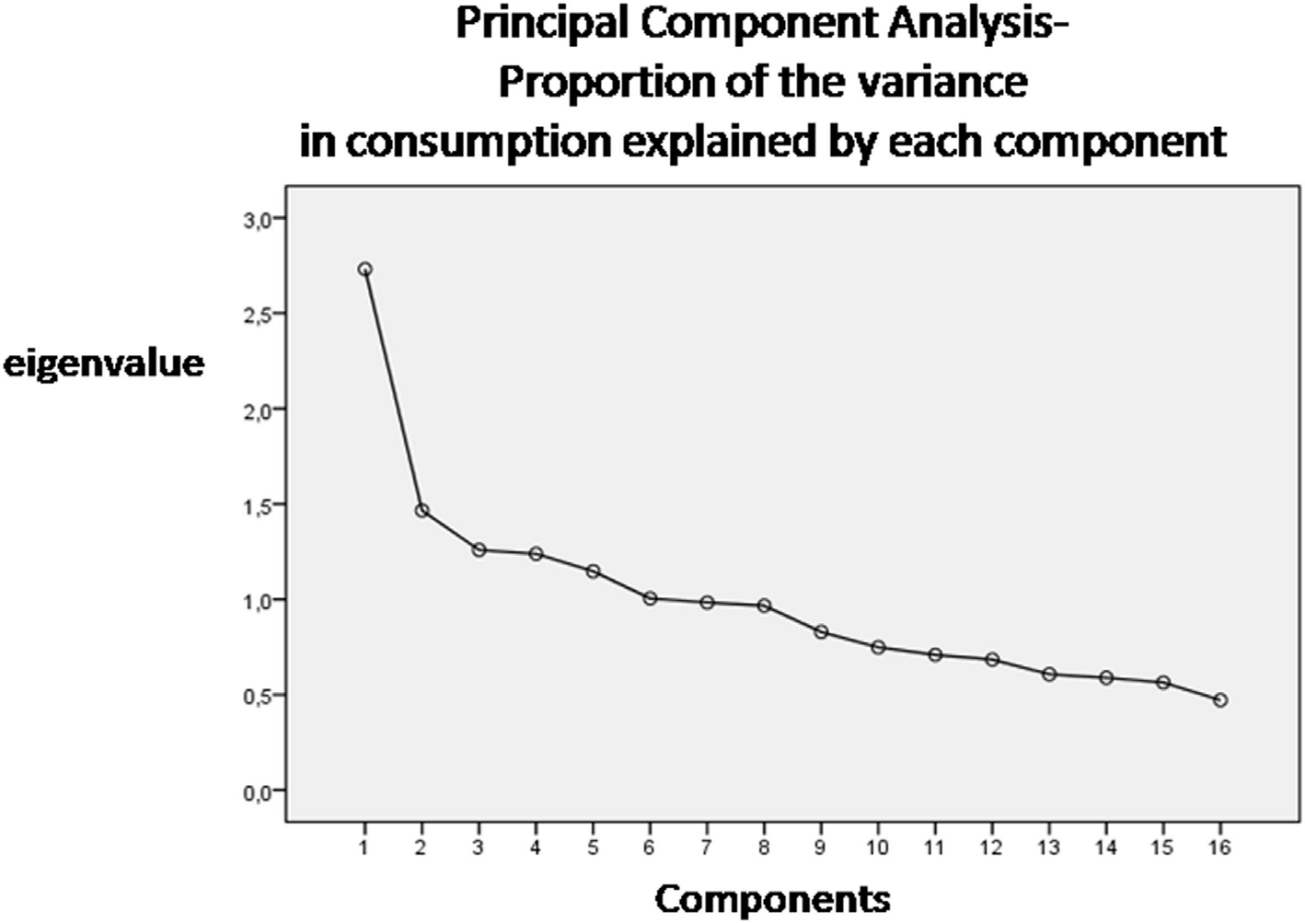
Screen plot of the eigenvalues–number of components (dietary patterns).

Six dietary patterns were identified (screen plot in Figure 1), with a cumulative explained variance of 55%, namely: Pattern 1 dietary pattern characterized by a high intake of meat, grains, olive oil, and potatoes; Pattern 2, characterized by a high intake of fish, vegetables and milk; Pattern 3, characterized by a high intake of cheese, cakes and fruit; Pattern 4, characterized by a high intake of cheese and animal-based fats; Pattern 5, characterized by a high intake of eggs, legumes, and wine and Pattern 6, characterized by a high intake of cakes, biscuits, and sugary drinks.

The following factors were correlated with WB-BMD: BMI (*r* = 0.14, *p* = 0.05), glucose (*r* = 0.24, *p* = 0.001), creatinine (*r* = 0.25, *p* = 0.001), statins (*r* = 0.15, *p* = 0.09), gender (*r* = 0.52, *p* = 0.001), antiosteoporotic agents (*r* = −0.16 and *p* = 0.065), and Pattern 1 (factor score as continuous variables, *r* = 0.19, *p* = 0.009), Pattern 2 (factor score, *r* = 0.14, *p* = 0.064), and Pattern 5 (factor score, *r* = 0.13, *p* = 0.096) (table not shown). No association with BMD was seen for any other dietary pattern.

The multivariate-adjusted mean WB-BMD across tertiles of all three dietary patterns is shown in Table 3. A significant and positive linear trend was observed for WB-BMD across increasing tertiles of dietary patterns (*P-*trend <0.05 for each). The highest tertile of both the Pattern 1 and 2 had a significantly higher WB-BMD than the lowest tertile after adjustment (model 2) for non-dietary factors and medications (Pattern 1: 1.021 ± 0.01 and 1.070 ± 0.01 g/cm^2^ for T1 and T3, respectively; *p* = 0.043; Pattern 2: 1.023 ± 0.01, and 1.081 ± 0.01 g/cm^2^ for T1 and T3, respectively; *p* = 0.003; Table 3). Table S2 in Supplementary Material reports the principal tertile characteristics within each dietary pattern. In particular, the highest tertile of the pattern 1 had a significantly higher grains, fish, and olive oil consumption than the lowest tertile (*p* < 0.001; *p* = 0.007 and *p* < 0.001, respectively); the highest tertile of the pattern 2 had a significantly higher grains, vegetables and olive oil consumption than the lowest tertile (*p* = 0.002; *p* < 0.001 and *p* < 0.001, respectively); the highest tertile of the pattern 5 had a significantly higher legumes, wine, and olive oil consumption than the lowest tertile (*p* < 0.001; *p* < 0.001 and *p* = 0.03, respectively) (Table S2 in Supplementary Material).

**Table 3:** Multivariate-adjusted mean WB-BMD across tertiles of dietary patterns- general linear model test.

Dietary pattern	Tertile 1 (low level) (*N* = 59)	Tertile 2 (medium level) (*N* = 59)	Tertile 3 (high level) (*N* = 59)	*P* for trend	*P*-post hoc analysis
**Food pattern 1**					

WB-BMD (g/cm^2^)	1.023 ± 0.12	1.022 ± 0.13	1.085 ± 0.10	0.002	I vs. III 0.021
					II vs. III 0.004
Model 1	1.010 ± 0.01	1.006 ± 0.01	1.086 ± 0.01	<0.001	I vs. III 0.001
					II vs. III <0.001
Model 2	1.021 ± 0.01	1.013 ± 0.01	1.070 ± 0.01	0.043	I vs. III 0.040
					II vs. III 0.019

**Food pattern 2**					

WB-BMD (g/cm^2^)	1.039 ± 0.10	1.020 ± 0.14	1.069 ± 0.11	0.031	II vs. III 0.031
Model 1	1.023 ± 0.01	1.013 ± 0.01	1.065 ± 0.01	0.054	II vs. III 0.022
Model 2	1.023 ± 0.01	1.000 ± 0.01	1.081 ± 0.01	0.003	I vs. III 0.018
					II vs. III 0.001

**Food pattern 5**					

WB-BMD (g/cm^2^)	1.030 ± 0.11	1.030 ± 0.12	1.057 ± 0.13	0.34	
Model 1	1.014 ± 0.01	1.032 ± 0.01	1.056 ± 0.01	0.19	
Model 2	1.021 ± 0.01	1.039 ± 0.01	1.044 ± 0.01	0.64	

*Model 1: BMI, glucose, and creatinine adjusted. Model 2: further adjustment for gender, current smoking and medications (antiresorptive agents/calcium/vitamin D supplementation; statins; antihypertensives; anticoagulants; oral antidiabetics)*.

*WB-BMD, whole-body bone mineral density; BMI, body mass index*.

Table S3 in Supplementary Material shows the multivariate-adjusted mean WB-BMD across tertiles of all dietary patterns by gender. In female, the highest tertile of the pattern 1 had a significantly higher adjusted WB-BMD than the lowest tertile (Table S3 in Supplementary Material). In male, the highest tertile of the pattern 2 had a significantly higher adjusted WB-BMD than the lowest tertile (Table S3 in Supplementary Material). We adjusted WB-BMD according with the variables which were significant different between gender (Table 2) and medications and current smoking.

### Dietary Patterns and Decreased Odds of Having Fractures

Pearson’s correlation was performed to identify which dietary pattern correlated with fractures. Only dietary Pattern 5 was correlated (*p* = 0.01, *r* = −0.16) (table not shown).

With the multinomial logistic regression analysis, a significantly lower adjusted OR for “Fractures” was estimated in individuals for the Tertile II (T2, OR = 0.20, *p* = 0.009) than those in the Tertile I (T1, OR = 0.21, *p* = 0.01) (T3 reference category) (OR medications, BMI, current smoking, and gender adjusted) (Table 4).

**Table 4:** Multinomial logistic regression analysis—“Fractures” being the dependent binary variable, adjusted predictions with 95% CI.

				C.I. 95%		

Dependent binary variable “Fractures”						

	B	SE	*p*	OR	LL	UL
Dietary pattern 5						
BMI	−0.019	0.04	0.68	0.98	0.892	1.078
Tertile 1	−1.545	0.609	0.011	0.213	0.065	0.703
Tertile 2	−1.567	0.599	0.009	0.209	0.064	0.675
Tertile 3	–	–	–	–	–	–
Medications	0.136	0.450	0.76	1.146	0.474	2.769
Gender	−0.795	0.539	0.14	0.452	0.152	1.298

*OR, odds ratio; CI, confidence interval; LL, lower limit; UL, upper limit*.

### Association between Dietary Patterns and Fractures Number

The following factors were correlated with “multiple fractures”: BMI (*p* = 0.06, r = −0.29) and dietary pattern 1 (*p* = 0.038, r = −0.32; table not shown).

In the logistic regression analysis (Table 5), dietary pattern 1 remained negatively associated with multiple fractures (B = −1.27, *p* = 0.03; OR = 0.28, CI 0.08–0.89; the highest adherence the lowest risk).

**Table 5:** Logistic regression analysis—dietary patterns and other factors associated with “multiple fractures” as binary variable (individuals with one fracture vs. those with more than one fracture).

				C.I. 95%		

Dependent variable						

Multiple fractures						

	B	SE	*p*	OR	LL	UL
Food pattern 1	1.27	0.59	0.032	0.28	0.08	0.89

*Excluded variables: BMI, gender, current smoking, medications; B, unstandardized coefficients; p, probability value; OR, odds ratio; C.I., confidence interval; LL, lower limit; UL, upper limit*.

No association with multiple fractures was seen for any other dietary patterns, BMI, and other potential confounding factors as gender, current smoking, and medications (Table 5).

## Discussion

Using the PCA, an approach that considers overall eating patterns, we identified 6 dietary patterns in 177 elderly individuals aged ≥65 years. However, only three patterns (Patterns 1, 2, and 5, characterized, as a whole, by a high intake of meat, grains, olive oil, potatoes, wine, legumes, and eggs) were correlated with WB-BMD. We found that the highest tertile of both the dietary pattern 1 and 2 had a significantly higher WB-BMD than the lowest tertile, after adjustment for non-dietary factors and medications (Table 3). In particular, in female the highest tertile of the pattern 1 had a significantly higher WB-BMD than the lowest tertile, while in male, the highest tertile of the pattern 2 had a significantly higher WB-BMD than the lowest tertile (Table S3 in Supplementary Material). Although all the highest tertiles of these dietary patterns were high in olive oil, they had a significantly higher grains and fish (pattern 1), grains and vegetables (pattern 2), and legumes and wine (pattern 5) than the lowest tertiles (Table S2 in Supplementary Material). Finally, in the whole population, the highest adherence to the dietary pattern 5 was associated with decreased odds of having fractures (Table 4) and adherence to the dietary pattern 1 was negatively associated with multiple fractures (Table 5).

The choice of different foods in the elderly has a significant impact on bone health (17, 20), but there are also studies with negative findings (21–23). Few studies have investigated the association between dietary patterns and WB-BMD in the elderly (24, 25). In one study, the authors enrolled only adult women while, in our study, we investigated both genders (24). In another study, a greater adherence to an overall healthy lifestyle including adequate physical activity was associated with better BMD at various sites among elderly Chinese individuals (25). Compared to the aforementioned studies, our study can, therefore, viewed as an original investigation.

We investigated the potential effects of dietary patterns on WB-BMD since several biases are systematically found in older populations over the age of 65 years with a DXA scan (5–7). Disk degeneration and fractures increase vertebral and/or hip BMD, aortic calcifications project additional minerals in the spine area. In one study, lumbar spine osteophytes affected 75% of men and 61.1% of women, and hip osteophytes affected 31.7% of men and 27.4% of women (7). Furthermore, bone loss predominantly regards the cortical bone in the elderly (10), and a strong association between WB- BMD and fractures was reported (9). However, aside from the accuracy in the diagnosis of osteoporosis (26), our study can be considered important as it could reveal a new approach to maintaining BMD.

Our results are, to some extent, confirmed by those reported in previous studies focusing on femoral BMD. In one study, adherence to a fruit, vegetable, and dairy pattern was associated with high femoral BMD and a low risk of fractures (3). The combined intake of vegetables, meat, fish, potatoes and wine has also been shown to be associated with a high femoral BMD in a population of middle-aged and elderly subjects (27). In this study two dietary patterns, similar to those involved in our study, were associated with a higher BMD: a “Traditional” pattern, characterized by a high intake of potatoes, meat and fat, and a “Health conscious” dietary pattern, characterized by a high intake of fruit, vegetables, poultry, fish, and alcohol (27). A dietary pattern including a high consumption of fish and olive oil, as in our pattern 1, was positively related to WB-BMD in adult women (24) confirming our results in women (Table S3 in Supplementary Material). In addition, in one study, both legumes and meat intake independently reduced the risk of hip fracture by 40–64% (28), partially confirming our findings (Tables 3 and 4).

In our study, males and females have the same amount of energy intake. Probably, women were significantly more likely to perceive themselves as susceptible to osteoporosis than male, perceiving greater benefits from nutrients intake (29).

Although our study was not designed to investigate the mechanisms underlying the association between dietary pattern and BMD or bone loss, our results highlight the important effect of a combination of nutrients on bone. The anti-inflammatory effect associated with an adherence to a dietary pattern including whole grains, olive oil, legumes, vegetables, and fish are well known (30), which could be crucial in preventing age- and menopause-related bone loss (31). A dietary pattern high in protein, as in the pattern 1 (highest tertiles = 68 g/die) and 2 (highest tertile = 77 g/die) has a protective effect on bone probably because the dietary protein intake increases the absorption of calcium in the intestine, the serum level of the anabolic hormone insulin-like growth factor 1 and decreases the serum parathyroid hormone (32).

Zinc, copper, manganese, and other micronutrients found in abundance in fruit and vegetables may also have an important role since they are known to be essential metallic cofactors for enzymes involved in the synthesis of various bone matrix components (33). The similarity in structure between isoflavones and estrogen together with the findings that isoflavones possess an estrogenic activity, as shown by several studies, provide the basis for speculation that isoflavones from legumes may promote bone health and reduce the risk of fracture (34).

Our finding, therefore, highlight the potential bone-preserving properties of specific dietary patterns in the elderly and should encourage the elderly to increase their weekly intake of specific food groups (for example, more than three serving of cereals and vegetables per day and three servings of meat and legumes per week).

### Strengths and Limitations

Our study has several strengths. We investigate the influence of medications and other non-dietary factors on the associations between dietary pattern and WB-BMD (Table 3). The use of a PCA to determine dietary patterns provides a realistic reflection of dietary patterns in a study population, and the six patterns identified in this study explain 55% of the overall variance. However, we also recognize various limitations. Residual confounding related to a general healthy lifestyle might still be present. PCA does not automatically provide the most optimal dietary pattern. Our sample size is relatively small and generalizability to other groups with different dietary patterns is not possible. Of course, with a cross-sectional study there is no evidence of a temporal relationship between exposure and outcome. However, few studies have investigated the association between dietary patterns and WB-BMD in the elderly. Thus, these results may suggest the hypotheses for a future, more complex, investigation. Finally, none of the bone turnover markers, which have a large day-to-day variations especially in patients with fractures (35), were assessed.

## Conclusion

This investigation emphasizes the possible key role of a dietary pattern, characterized by a high intake of meat, grains, olive oil, and potatoes and a dietary pattern characterized by a high intake of fish, vegetables, and milk in preserving WB-BMD. The highest adherence to the dietary pattern 5 characterized by a high intake in legumes, eggs, and wine, was also associated with decreased odds of having fractures and the high adherence to the dietary pattern 1 was associated with a low number of fractures.

## Ethics Statement

This study protocol was approved by the local ethics committee at the “Mater Domini” University Hospital in Catanzaro, Italy (projects codes 2011.48) and approved by the Italian Ministry of Health with written informed consent from all subjects. The investigation conforms to the principles outlined in the Declaration of Helsinki.

## Author Contributions

TM and AP were responsible for study design, data analysis, manuscript writing. CC, EM, AF, and YF were responsible for data collection and integrity of data. CC, AF, and YF were responsible for enrollment. EM, AF, and DB were responsible for anthropometric and nutritional measurements. MG, DPF, SR, and EG were responsible for laboratory assessment. All authors approved final manuscript.

## Conflict of Interest Statement

The authors CC, EM, YF, AF, DB, MG, DF, EG, SR, AP, and TM declare that they have no competing interests. The reviewer AS and handling Editor declared their shared affiliation.

## Abbreviations

BMI, body mass index; WB-BMD, whole-body bone mineral density; DXA, dual X-ray absorptiometry; 25OH-vitamin D, 25-hydroxy-vitamin D; PCA, principal components analysis; SPA, sufficient levels of physical activity.
