# Supplementary material for: Dietary Patterns and Fractures Risk in the Elderly
Source: Front Endocrinol (Lausanne). 2017 Dec 13;8:344. doi: 10.3389/fendo.2017.00344 (PMC5770658; doi:10.3389/fendo.2017.00344)
Supplement: Supplementary file 1 [file Data_Sheet_1.PDF]

Suppl. Table 1- Participants' demographic, anthropometric, clinical and energy and food groups intak characteristics in the population according to gender

Variables	Females (n=112)	Males (n=65)	p-value
Age (years)	69±4	70±4	0.045
BMI (Kg/m^2)	28.7±4	28.4±4	0.53
BMD (g/cm^2)	69±4	69±4	<0.001
Total body T-score	-1.2±1.5	-0.5±1.0	<0.001
Total body Z-score	-0.1±1.0	0.1±0.8	0.16
Nutrients			
Energy Intake (kcal)/day	1858±433	1884±478	0.69
Carbohydrates (g/day)	216±57	216±64	0.95
Lipids (g/ day)	71±22	73±23	0.42
Protein (g/day)	77±22	76±23	0.76
Food groups			
Cereals (g)*	101±37	124±55	0.001
Legumes (g)*	10±13	10±9	0.97
Potatoes (g)*	11±13	11±14	099
Vegetables (g)*	154±86	156±90	0.87
Fruit (g)*	184±106	198±124	0.40
Milk (g)*	75±61	61±61	0.11
Cheeses (g)*	33±24	36±30	0.46
Eggs (g)*	6±6	8±14	0.31
Meat (g)*	41±26	49±27	0.034
Fish (g)*	31±25	37±35	0.18
Wine (g)*	23±44	67±75	<0.001
Drinks (g)*	10±27	13±43	0.49
Virgin Olive oil (g)*	19±9	20±7	0.70
Animalfats/margarines (g)*	0.5±0.2	0.2±0.5	0.14
Cookies (g)*	5±8	6±10	0.83
Cakes/pies (g)*	20±19	20±17	0.90

Note. *adjusted for 1000/kcal. BMI = body mass index;BMD = bone mineral density.

Suppl. Table 2 Tertiles characteristics within each dietary pattern.

Variables	Tertile 1 (Low level)	Tertile 2 (medium level)	Tertile 3 (high level)	*p-value*	*P-Post-Hoc Analysis*
Food pattern 1					
Cereals (g)*	82±31	105±36	139±48	<0.001	I vs II 0.001<br>I vs III <0.001<br>II vs III <0.001
Legumes (g)*	10±14	9±9	9±9	0.98	
Vegetables (g)*	152±92	150±74	160±93	0.75	
Fish (g)*	25±23	33±31	40±29	0.007	I vs III 0.002
Wine (g)*	43±56	36±66	37±59	0.74	
Olive oil (g)*	14±5	18±6	24±9	<0.001	I vs II 0.003<br>I vs III <0.001<br>II vs III <0.001
Food pattern 2					
Cereals (g)*	96±42	107±44	123±47	0.002	I vs III 0.001<br>I vs III 0.032
Legumes (g)*	9±10	8±9	12±13	0.12	
Vegetables (g)*	117±67	156±78	189±98	<0.001	I vs II 0.006<br>I vs III <0.001<br>II vs III 0.018
Fish (g)*	37±33	30±27	31±24	0.23	
Wine (g)*	38±59	47±71	30±49	0.26	
Olive oil (g)*	16±7	19±7	22±9	0.001	I vs III <0.001<br>II vs III 0.042
Food pattern 5					
Cereals (g)*	112±46	102±47	112±42	0.34	
Legumes (g)*	3±3	7±5	19±15	<0.001	I vs II 0.024<br>I vs III <0.001<br>II vs III <0.001
Vegetables (g)*	143±89	149±89	170±80	0.15	
Fish (g)*	36±28	27±26	35±31	0.12	
Wine (g)*	7±15	19±26	89±79	<0.001	I vs III <0.001<br>II vs III <0.001
Olive oil (g)*	17±6	19±9	21±8	0.032	I vs III 0.010

*Note.**adjusted for 1000/kcal.

Suppl. Table 3- Multivariate-adjusted mean WB-BMD across tertiles of dietary patterns according to gender-General linear Model test

Dietary patterns (Female)	Tertile 1 (low level) (N=37)	Tertile 1 (medium level) (N=37)	Tertile 1 (high level) (N=38)	*P for trend*	*P-Post-Hoc Analysis*
Food pattern 1					
BMD (g/cm^2)	0.983±0.10	0.972±0.12	1.028±0.09	0.085	II vs III 0.030
BMD adjusted	1.016±0.02	1.013±0.02	1.087±0.02	0.08	**I vs III 0.009** II vs III 0.006
Food pattern 2					
BMD (g/cm^2)	1.004±0.10	0.955±0.12	1.014±0.10	0.036	I vs II 0.050 II vs III 0.016
BMD adjusted	1.036±0.02	1.002±0.02	1.078±0.02	0.023	II vs III 0.006
Food pattern 5					
BMD (g/cm^2)	0.984±0.09	0.993±0.11	0.993±0.12	0.92	
BMD adjusted	1.028 ±0.02	1.038±0.02	1.050±0.02	0.72	

Dietary patterns (Male)	Tertile 1 (N=22)	Tertile 2 (N=22)	Tertile 3 N=21)	*P for trend*	*P-Post-Hoc Analysis*
Food pattern 1					
BMD (g/cm^2)	1.109±0.13	1.116±0.09	1.138±0.09	0.58	
BMD adjusted	1.111±0.03	1.129±0.03	1.135±0.02	0.76	
Food pattern 2					
BMD (g/cm^2)	1.093±0.09	1.117±0.12	1.161±0.09	0.07	I vs III 0.025
BMD adjusted	1.088±0.03	1.123±0.02	1.162 ±0.02	0.11	**I vs III 0.041**
Food pattern 5					
BMD (g/cm^2)	1.130±0.09	1.097±0.11	1.139±0.10	0.35	
BMD adjusted	1.124±0.03	1.139±0.02	1.117±0.03	0.80	

Note. (female) WB-BMD adjusted for current smoking and medications ; (male) WB-BMD Adjusted for age, current smoking and medications. BMD = bone mineral density.
